# Evidence for high-performance suction feeding in the Pennsylvanian stem-group holocephalan *Iniopera*

**DOI:** 10.1073/pnas.2207854119

**Published:** 2023-01-17

**Authors:** Richard P. Dearden, Anthony Herrel, Alan Pradel

**Affiliations:** ^a^CR2P, Centre de Recherche en Paléontologie–Paris, Muséum National d’Histoire Naturelle, Sorbonne Université, Centre National de la Recherche Scientifique, CP 38, Paris Cedex 05, F75231, France; ^b^School of Geography, Earth and Environmental Sciences, University of Birmingham, Edgbaston, Birmingham, B15 2TT, United Kingdom; ^c^UMR 7179 MECADEV (Mécanismes adaptatifs & Evolution), Département Adaptations du Vivant, Muséum National d’Histoire Naturelle, Sorbonne Université, Centre National de la Recherche Scientifique, CP 38, Paris Cedex 05, F75231, France

**Keywords:** holocephalan, Carboniferous, suction feeding, iniopterygian, Pennsylvanian

## Abstract

The Carboniferous (358.9 to 298.9 Ma) saw the emergence of marine ecosystems dominated by modern vertebrate groups, including abundant stem-group holocephalans (chimaeras and relatives). Compared with the handful of anatomically conservative holocephalan genera alive today—demersal durophages all—these animals were astonishingly morphologically diverse, and bizarre anatomies in groups such as iniopterygians hint at specialized ecological roles foreshadowing those of the later, suction-feeding neopterygians. However, flattened fossils usually obscure these animals’ functional morphologies and how they fitted into these important early ecosystems. Here, we use three-dimensional (3D) methods to show that the musculoskeletal anatomy of the uniquely 3D-preserved iniopterygian *Iniopera* can be best interpreted as being similar to that of living holocephalans rather than elasmobranchs but that it was mechanically unsuited to durophagy. Rather, *Iniopera* had a small, anteriorly oriented mouth aperture, expandable pharynx, and strong muscular links among the pectoral girdle, neurocranium, and ventral pharynx consistent with high-performance suction feeding, something exhibited by no living holocephalan and never clearly characterized in any of the extinct members of the holocephalan stem-group. Remarkably, in adapting a distinctly holocephalan anatomy to suction feeding, *Iniopera* is more comparable to modern tetrapod suction feeders than to the more closely related high-performance suction-feeding elasmobranchs. This raises questions about the assumed role of durophagy in the evolution of holocephalans’ distinctive anatomy and offers a rare glimpse into the breadth of ecological niches filled by holocephalans in a pre-neopterygian world.

## Significance

Suction is an especially effective way of feeding underwater, and adaptations to enhance it have evolved numerous times in jawed vertebrates. The only major living jawed vertebrate group including no specialist suction feeders is chimaeras, a handful of anatomically conservative fish species that feed on hard-shelled prey. Contrastingly, in the Carboniferous (359 to 299 Ma), diverse chimaeras formed a prominent part of aquatic ecosystems. Here, we use three-dimensional–preserved fossils of one of these Carboniferous chimaeras to reconstruct its cranial muscles and argue that it had the forward-facing mouth and expandable pharynx characteristic of high-performance suction feeders. This suggests that in the Carboniferous, some chimaeras were suction feeders in the water column, an ecological niche since monopolized by neopterygian fishes.

Aquatic jawed vertebrates are uniquely adept at suction feeding, namely, sucking water and prey into the mouth by expanding the volume of the oral cavity to generate a pressure differential between it and the external environment (1). Effective suction feeding has the following two main anatomical requirements: a laterally restricted, anteriorly facing mouth aperture and a means of rapidly increasing the volume of the oral cavity (1, 2). The visceral arches of gnathostomes (jawed vertebrates) make them especially well-suited to satisfying these requirements compared with jawless taxa, and diverse adaptations to enhance suction feeding and create high-performance suction-feeding systems have evolved in elasmobranchs, sarcopterygians, and actinopterygians, which are three of the four major gnathostome divisions (1). Suction-feeding elasmobranchs, both extant [e.g., bamboo sharks (3)] and extinct [the Carboniferous stem-group elasmobranch *Tristychius* (4)] use labial cartilages to demarcate the oral margin and the hyoid arch and pectoral girdle to expand the oral cavity to create suction and move prey down the long pharynx. Among sarcopterygians, coelacanths suction feed with a piscine anatomy (5), while tetrapods, including salamanders (6), turtles (7, 8), mammals (9), and frogs (10), expand the pharynx using the hyoid and pectoral skeletons, in some cases delimiting the mouth laterally with fleshy lobes. The apogee of living jawed vertebrate suction feeding is in neopterygian actinopterygians, which demarcate the oral opening and expand the pharynx using specialized dermal skull bones in combination with epaxial/hypaxial muscles (11), a system that evolved in the late Permian (12) and has led them to dominate aquatic vertebrate faunas ever since. The main exception to the ubiquity of suction feeding in gnathostomes is the fourth major division of jawed vertebrates, namely, holocephalans. Instead of suction feeding, all living holocephalans use a highly derived arrangement of labial cartilages, jaws, cranial muscles, and hypermineralized toothplates to feed on benthic, often hard-shelled, prey (13–15).

In this paper, we use digital three-dimensional (3D) methods to characterize the functional morphology of *Iniopera*, an iniopterygian stem-group holocephalan from Pennsylvanian of the United States. Iniopterygians are known from marine faunas from the Serpukhovian through to the Kasimovian (∼330.9 to 303.9 Ma) (16–19). Although iniopterygians have been placed on the chondrichthyan stem-group (20, 21), in more recent phylogenetic analysis, they have been consistently recovered as stem-group holocephalans on the basis of key shared traits (22–25). Despite this, iniopterygians had a highly peculiar anatomy unlike that of living holocephalans (16). Among iniopterygians, *Iniopera* is the only taxon, and one of very few stem-group holocephalans, known from 3D-preserved remains, which include the skull, jaws, shoulder girdle, pharyngeal skeleton, and brain (26–29). *Iniopera* has been interpreted as being durophagous (16, 28) and shares with living holocephalans inferred adaptations to durophagy including a holostylic neurocranium (i.e., upper jaws and braincase fused), a relatively anteriorly placed jaw articulation, and a lower jaw with a fused symphysis. However, *Iniopera* lacks key adaptations of living holocephalans to durophagy including toothplates on the mandibular arch and a vaulted neurocranium, as well as adaptations to benthic feeding including large labial cartilages, and an anteroventrally oriented mouth. Here, we use 3D digital models of the skull, branchial, hyoid, and pectoral skeleton of *Iniopera* to explore its functional morphology. Our results suggest that rather than being durophagous, *Iniopera* was a high-performance suction-feeder.

## Results

### Overview of Cranial Muscle Reconstruction.

**Fig. 1.**
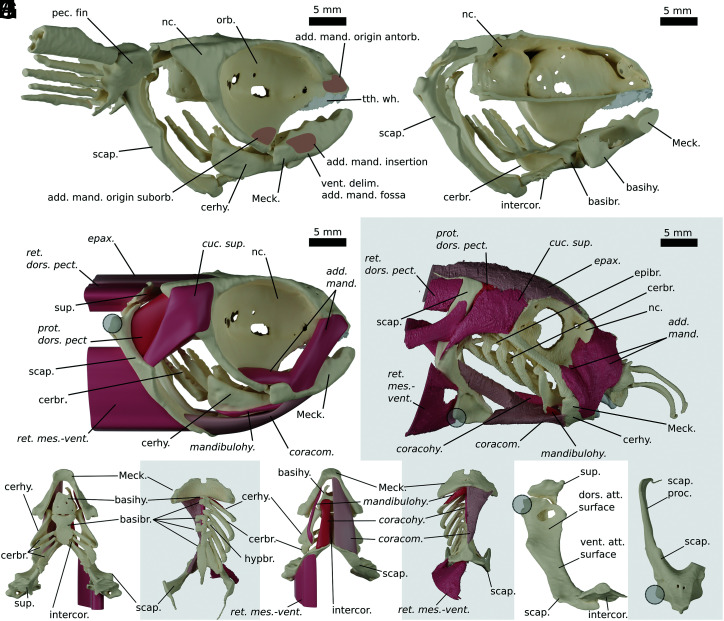
Reconstruction of muscles in *Iniopera* (white background) compared with the extant holocephalan *Callorhinchus* (gray background). (*A* and *B*) The skeleton of *Iniopera* reconstructed in (*A*) right lateral view and (*B*) bisected in right lateral view to show branchial and pectoral skeleton. (*C* and *D*) *Iniopera* (*C*) and *Callorhinchus* (*D*) in right lateral view. (*E* and *F*) The ventral pharyngeal skeleton of *Iniopera* (*E*) and *Callorhinchus* (*F*) in dorsal view. (*G* and *H*) The ventral pharyngeal skeleton of *Iniopera* (*G*) and *Callorhinchus* (*H*) in ventral view. (*I* and *J*) The right shoulder girdle of *Iniopera* (*I*) and *Callorhinchus* (*J*) in anterior view. Gray circle shows the position of pectoral fin articulation on scapulocoracoid. Abbreviations: add. mand., adductor mandibularis muscle; antorb., antorbital; basihy., basihyal; cerbr, ceratobranchials; cerhy, ceratohyal; coracohy., coracohyoideus muscle; coracom., coracomandibularis muscle; cuc. sup. cucullaris superficialis muscle; dors. att. surface, dorsal attachment surface of anterior scapulocoracoid; epax., epaxialis muscles; epibr., epibranchials; hypbr., hypobranchials; intercor., intercoracoid element; mandibulohy., mandibulohyoideus muscle; Meck., Meckel’s cartilage; nc., neurocranium; orb., orbit; pec. fin., pectoral fin; prot. dors. pect., protractor dorsalis pectoralis muscle; ret. dors.-pect., retractor dorsalis pectoralis muscle; ret. mes.-vent., retractor mesioventralis muscle; scap., scapulocoracoid; scap. proc., scapular process; suborb., suborbital; sup. suprascapular element; tth. wh., tooth whorl; vent. att. surface, ventral attachment surface of anterior scapulocoracoid; vent. delim., ventral delimitation. As noted in *SI Appendix*, the retractor mesioventralis in *Iniopera* may be hypaxial muscles.

The attachment areas on the skull of *Iniopera* are broadly consistent with the arrangement of cranial muscles in living holocephalans like *Callorhinchus* (Fig. 1 and *SI Appendix*, Figs. S1 and S2). Like crown-group holocephalans, the skull of *Iniopera* is holostylic and has a close relationship with the pectoral girdle (26, 28). We interpret a large part of the mandibular adductor muscle in *Iniopera* to have had an origin in the antorbital fossa (Fig. 1), like the anterior mandibular adductor of crown-group holocephalans, but with a relatively smaller origin and inserted at a more oblique angle on the mandible (Fig. 1 *A* and *C*). In the holocephalan crown-group, a much smaller posterior mandibular adductor also inserts on the suborbital ridge and preorbital fascia, which in chimaerids is reduced to a small patch of muscle fibers (13, 30). It seems likely that there was also a posterior part of the mandibular adductor in *Iniopera,* which inserted in the bottom part of the orbit (30). Ventrally delimited fossae on the Meckelian cartilages (Fig. 1*A*) suggest that the mandibular adductor inserted directly on the mandible as in shark-like chondrichthyans, rather than into a tendinous submandibular “sling” like living holocephalans (13, 14, 30). The attachment area of the epaxial muscles on the neurocranial roof is small compared with *Callorhinchus* (Fig. 1 *A* and *B*), but enormous fossae on either side of the foramen magnum would have provided insertions for muscles homologous or analogous with the m. (musculus) protractor dorsalis pectoralis of *Callorhinchus* (Fig. 1 *A, C, and D*).

Unlike living holocephalans, the pectoral girdle of *Iniopera* had dorsally located pectoral fins, suprascapular elements, and a separate intercoracoid element that articulated with the scapulacoracoids via well-developed articular fossae as well as with the bottom of the basibranchial (28, 29). The large posterior fossae on the neurocranium, together with large attachment surfaces on the basibranchial skeleton and scapulocoracoids, suggest that the shoulder girdle of *Iniopera* had a stronger muscular connection with the neurocranium and basibranchial skeleton than is found in living holocephalans. The basihyal and basibranchial skeletons are markedly robust in *Iniopera* and far larger proportionately than their equivalents in living holocephalans (Fig. 1 *C*–*F*), and while the insertion areas for the coracomandibularis are relatively smaller in *Iniopera* than in *Callorhinchus*, the coracohyoideus insertion is much larger, suggesting that abduction of the hyoid arch played a major role in feeding. The large ceratohyal flange seems likely to have acted as an insertion for an m. mandibulohyoideus like that of living holocephalans but with a larger attachment area (Fig. 1 *C*, *D*, and *E*) (29, 30). Notably, the ceratohyal in *Iniopera* does not appear to be in series with the branchial arches as in living holocephalans and has a far more robust connection with the basihyal (Fig. 1 *A*–*E*), suggesting it played a more active role. In *Iniopera*, the main attachment surface for muscles on the scapulocoracoid is below the fin articulation rather than above it on the scapular process as in *Callorhinchus* (Fig. 1 *I* and *J*); in *Iniopera*, this surface is broad and oriented anteroposteriorly, rather than narrow and oriented mediolaterally like in *Callorhinchus*, and ventral parts of the scapulocoracoids would have provided origins for some combination of coracomandibularis/hyoideus/branchialis muscles (Fig. 1 *G* and *H*). We interpret the large fossae on the anterodorsal surface of the scapulocoracoids as providing origins for the muscles inserting on the rear of the neurocranium (Fig. 1*G*) analogous to the m. protractor dorsalis pectoralis in *Callorhinchus* (although ventral relative to the fin articulation). The more laterally oriented face of this surface probably also provided an origin for fin adductor muscles (Fig. 1*G* and *SI Appendix*, Fig. S2). Similarly large surfaces are present posteroventrally for the attachment of the m. retractor mesioventralis pectoralis or the hypaxial equivalent. The proportions of the skeleton suggest that the orobranchial cavity was large compared with the parabranchial cavity, like in living chimaeras (29, 31). Full details of the skeletal models and a full justification for muscle placement are given in the *SI Appendix*, SI text.

### Modeling Functional Morphology.

**Fig. 2.**
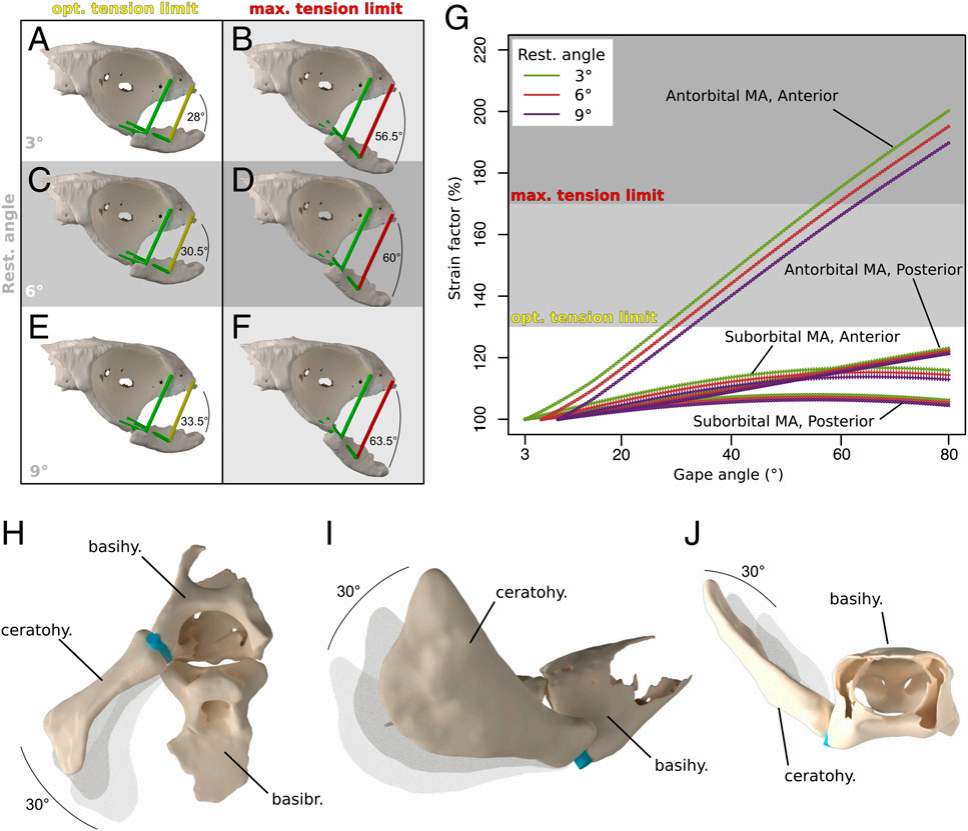
Estimates of constraints on the movement of the visceral skeleton in *Iniopera*. (*A*–*G*) Constraints on gape imposed by muscle extension, with mandibular adductor muscles modeled as tubes. (*A* and *B*) From a resting position of 3°. (*C* and *D*) From a resting position of 6°. (*E* and *F*) From a resting position of 9°. (*A*–*C*) Show the gape at the upper limit of the optimal tension of the mandibular adductor muscles. (*D*–*F*) Show the gape at the maximum extension limit of the mandibular adductor muscles. (*G*) Strain factor as a percentage of muscle extension plotted against gape angle for data from the three resting angles. (*H*–*J*) Constraints on movement of the ceratohyal as estimated in range of motion analysis. Blue cylinder represents location and angle of articulation between ceratohyal and basihyal, solid ceratohyal represents furthest possible abduction of ceratohyal, with paler copies representing original position and halfway point. Abbreviations: basibr., basibranchial; basihy., basihyal; ceratohy., ceratohyal; max. maximum; MA, mandibular adductor; opt., optimal; rest., resting.

**Fig. 3.**
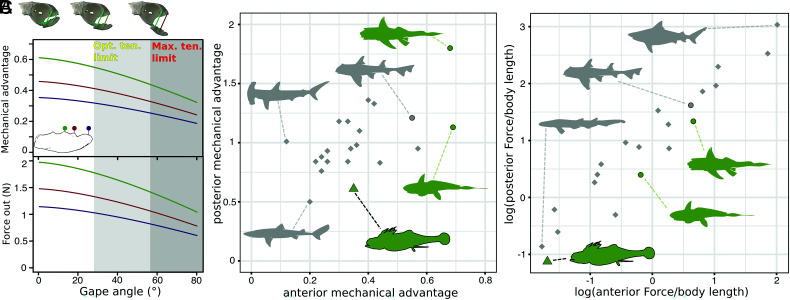
Mechanical advantage and estimated output force in *Iniopera*, compared with extant chondrichthyans. (*A*) Mechanical advantage (*Top*) and estimated force out (*Bottom*) through the jaw closing cycle for *Iniopera* for three points along the inferred tooth row, namely, posterior (green), midpoint (red), and anterior (purple). Ranges for the optimal tension limit of the mandibular adductor and the maximum tension limit use the estimate based on a resting angle of 3 degrees. (*B*) Anterior vs. posterior mechanical advantage of the jaws of *Iniopera* compared with extant chondrichthyans. (*C*) Anterior vs. posterior estimated output force of the jaws of *Iniopera* compared with extant chondrichthyans, logged relative to body length. A key to *B* and *C* is as follows: gray points, elasmobranchs; green points, holocephalans; circles, durophages; diamonds, nondurophages; triangle, *Iniopera*. Silhouettes from Phylopic all under a Public Domain Mark 1.0 license: *Scyliorhinus*, uploaded by Birgit Lang; *Squalus* uploaded by M Kolmann; *Hydrolagus* and *Chimaera*, uncredited. Other silhouettes were made by Richard P. Dearden from images in Wikimedia Commons (*Carcharhinus* and *Sphyrna*) and from Zangerl and Case (16) (*Iniopera*). Abbreviations: ten., tension, otherwise as in Fig. 2.

The skull of *Iniopera* sp. lacks key adaptations to demersal durophagy present in living holocephalans. Using our reconstruction and a 3D modeling approach that estimates the optimal and maximum tension limits of the mandibular adductor muscles (32), the maximum possible gape in *Iniopera* would have been between 56.5° and 63.5° and the mandibular adductor would have performed optimally up to a gape of between 28° and 33.5° (Fig. 2). Combined with its anterodorsal orientation and the lack of labial cartilages, this makes the mandible unsuited to scooping prey off the seafloor as in living holocephalans (15). Moreover, the mandibular adductor’s insertion at the posterior end of the mandible leads to a low mechanical advantage on the jaw, which is much lower than that in living durophagous chondrichthyans (Fig. 3 *A* and *B* and *SI Appendix*, Fig. S5*C*) (14). Based on this, we estimate that the maximum force that could have been produced is 3.94 N at the posterior end of the dentition (Fig. 3 *A* and *C*), which is low compared with living chondrichthyan durophages (Fig. 3*C* and *SI Appendix*, Fig. S5). *Iniopera*’s tooth whorls, blunter than those of *Sibyrhynchus*, have been cited as evidence of durophagy (16); however, they are unlike the dentition of any living chondrichthyan durophage and are concentrated anteriorly in the part of the gape with the lowest mechanical advantage. Combined with the lack of living holocephalans’ structural adaptations to the high forces of durophagy, such as a submandibular sling and cranial vaulting (14), *Iniopera* is unlikely to have been a durophage.

Rather, evidence from the anatomy of *Iniopera* and our reconstruction is consistent with *Iniopera* having been a high-performance suction feeder. An effect of the antorbital origin of the mandibular adductor muscles is that the mouth opening would have been small and anteriorly oriented, a characteristic of high-performance suction feeders (1). This interpretation is supported by the anterior position and exclusively anteroposterior orientation of the tooth whorls of *Iniopera* (and other iniopterygians) that would have faced this opening (16). The strong functional connection between the shoulder girdle of *Iniopera* and the hyoid arch has been linked to the accordion ventilation of living holocephalans (29), but the extremely large size of the hyoid and basibranchial skeleton relative to those of crown-group holocephalans (Fig. 1 *C*–*F*) instead recalls hyoid elements in high-performance suction-feeding gnathostomes (3, 6–8, 11). The large coracohyoideus linking the basihyal and shoulder girdle as well as the link between the intercoracoid and basibranchial elements would have allowed a strong contraction of the ventral pharynx. The large muscles linking the neurocranium and shoulder girdle dorsally would have had the effect of anchoring the scapulocoracoid dorsally while the large hypaxial muscles pulled the ventral shoulder girdle posteriorly, as in the suction-feeding cycle of largemouth bass and bamboo sharks (3, 11). This in turn would have pulled the bottom of the pharynx ventrally and posteriorly, due to the musculoskeletal connections between the two (29). The comparatively small epaxial attachments on the neurocranium suggest that that a movement of the neurocranium to expand the pharynx, as in actinopterygians and coelacanths (5, 11), was minimal. The heart in *Iniopera* likely lays over the intercoracoid, with the conus arteriosus and ventral aorta passing anteriorly before splitting into the afferent hyoid arteries within the basibranchial (29); a notable result of our model is that this ventral vasculature would have been swung posteriorly and anteriorly with the movement of the shoulder girdle.

The hyoid arch is an important part of the suction-feeding system in living jawed vertebrates (1). In *Iniopera*, the only preserved parts of the hyoid arch are the basihyal and ceratohyal; the morphology of the hyomandibula and whether or not the hyoid arch articulated with the braincase are unknown (26, 29). However, the movement of the ceratohyal relative to the basihyal provides a constraint on ceratohyal function. Our range-of-motion analysis of this joint suggests that the ceratohyal was able to move outward by a maximum of 30 degrees (Fig. 2 and *SI Appendix*, Fig. S3) and would have swung anterolaterally relative to the basihyal. This motion would be consistent with the ceratohyal being connected to the braincase, by either a cartilage (hyomandibula) or ligament, and being forced outward and forward as the pharyngeal floor is pulled back, as in living suction-feeding fishes.

**Fig. 4.**
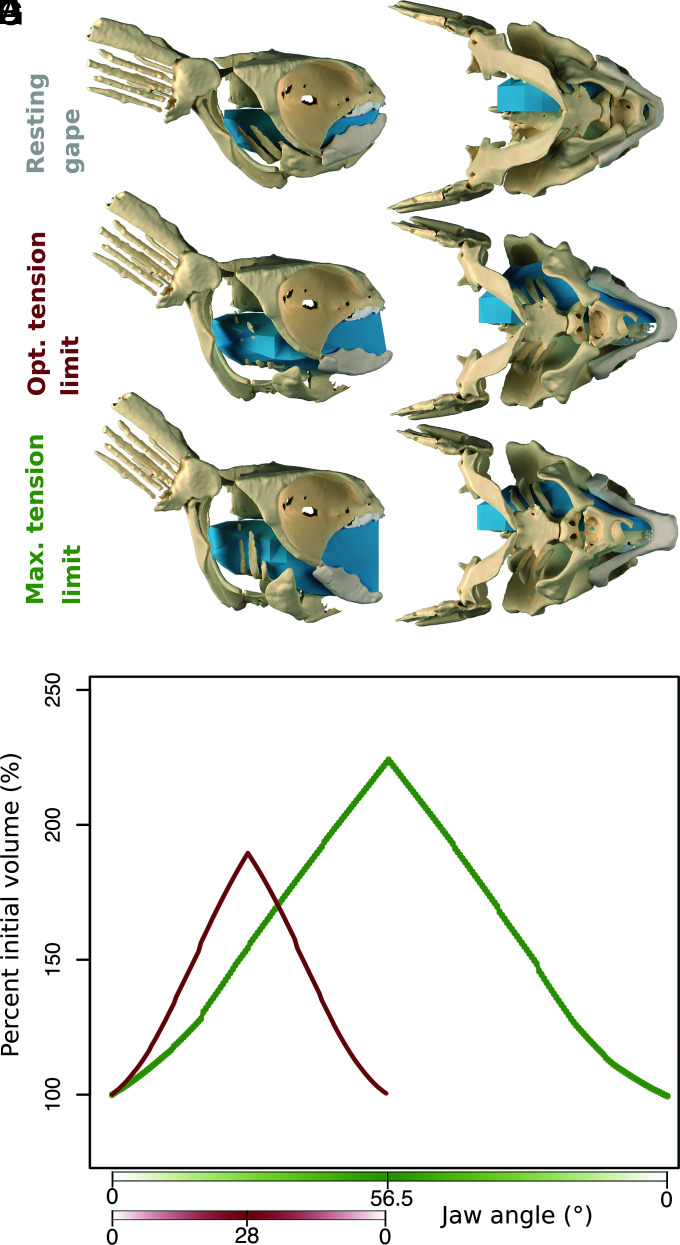
Estimate of pharyngeal expansion in *Iniopera* based on animated model with the jaw opening to the upper limit of the optimal tension of the mandibular adductor muscle (red) and the maximum tension limit of the extension range of the mandibular adductor (green) using gapes established for a resting angle of 3°. (*A*–*F*) Model shown at closed (*A*), optimal tension limit (*B*), and maximum tension limit (*C*) in (*A*, *C*, and *E*) right lateral view and (*B*, *D*, and *F*) ventral view. (*G*) Graph of jaw angle for the upper limit of the optimal extension range of the mandibular adductor extension and the maximum limit of mandibular adductor extension plotted against percentage of initial volume.

Our 3D modeling based on our reconstruction shows that this system could have acted to draw the floor of the pharynx posteroventrally as the pectoral girdle was pulled posteriorly (Fig. 4). Based on this movement and the lateral flaring of the ceratohyals, we estimate that *Iniopera* could have expanded its buccal cavity and pharynx by 88.7% (at the limit of the optimal tension of mandibular adductor extension) and 124.3% (at the maximum limit of mandibular adductor extension) from the lowest resting gape position (Fig. 4). These are probably overestimates, due to the nonsequential nature of our model, but illustrate that the system would have been capable of generating a significant increase in volume.

## Discussion

Adaptations to facilitate high-performance suction feeding have convergently evolved numerous times in the gnathostome total-group (1) but *Iniopera* is the first holocephalan in which there is clear evidence for this feeding mode. This is a relatively phylogenetically remote example of convergence that surprisingly is more similar to living tetrapods than to living high-performance suction-feeding fishes. All living chondrichthyan high-performance suction feeders are elasmobranchs, which have evolved the feeding mode several times independently (33). Elasmobranch suction feeders use labial cartilages to constrict an anteriorly oriented mouth opening and enlarge the mouth cavity by depressing the jaw and hyoid, and they use the pectoral girdle to move prey down the long pharynx (3), a strategy that was also present in the Mississippian stem-group elasmobranch *Tristychius* and likely also some hybodonts (4). Actinopterygian suction feeders have short subcranial pharynxes like *Iniopera* and also use a linked cranial, pectoral, and hyoid skeleton to increase the size of the buccal cavity (11). However, this comprises a complicated linkage system involving dermal bones, which are also used to delineate the mouth opening, and also includes a large epaxial component pulling the head back, which are both absent in *Iniopera* (11, 34). The clearest living analog to *Iniopera* instead lies in sarcopterygian tetrapod suction feeders such as the matamata turtle (7, 8), *Pipa* frog (10), and aquatic salamanders (6). Although the anatomy of these animals differs from one another, these taxa have evolved high-performance suction feeding under the constraint of a fused upper jaw and braincase, a short pharynx, and no large dermal plates by using an enlarged hyoid arch and pectoral girdle to expand the pharynx. *Iniopera* seems to have convergently evolved a similar functional morphology, although notably, unlike tetrapods (with the exception of some paedomorphic salamanders), water would have flowed unidirectionally into the mouth and out through the gill openings (1). Although we do not attempt to model the activity sequence here, *Iniopera* presumably expanded its pharynx in an anterior-to-posterior sequence as in living suction-feeding gnathostomes, drawing water and prey into the mouth and using its anteriorly aligned tooth whorls to secure prey (1, 35).

High-performance suction feeding in *Iniopera* had its basis in a distinctively holocephalan anatomy. Holostylic, vaulted neurocrania, large pre- and suborbital mandibular adductor muscles, and jaws with a high mechanical advantage are key components of crown-group holocephalans’ adaptations to durophagy (14, 15). Parts of this anatomical suite, which has been linked to the evolution of durophagy (36), are present in Carboniferous stem-group holocephalans with dentitions at least somewhat adapted to crushing—for example in *Chondrenchelys* and *Helodus*, which have holostylic neurocrania and pre- or suborbital mandibular adductor origins (37–39). *Iniopera* has key components of this system—a holostylic jaw suspension with mandibular adductors with pre- and suborbital origins—and yet is unsuited to durophagy. It is likely that other iniopterygians displayed a similar condition, at least in other Sibyrhynchidae that have similar visceral skeletons, dentitions, and neurocrania to *Iniopera* (16). In the Iniopterygidae, which had free palatoquadrates and are only known from flattened specimens (16, 17, 19), the situation is less clear, although reconstructions of the palatoquadrate imply that a posteriorly restricted mandibular adductor attachment would have had a very low mechanical advantage (e.g., figure 5 in ref. 17). This raises the possibility that anatomies associated with durophagy such as pre- or suborbital attachment of the mandibular adductors preceded holostyly and did not necessarily evolve alongside durophagy. Conversely, an intercoracoid, a key component of *Iniopera*’s suction-feeding anatomy absent in living holocephalans, is present in the durophagous Jurassic putative chimaeroid *Ischyodus* (28, 40). Holostyly appears to have evolved several times convergently in the holocephalan total-group, as well as in iniopterygians; the putative stem-holocephalan Eugeneodontidae includes taxa with palatoquadrates fused to [e.g., *Ornithoprion* (41)] and free from [e.g., *Helicoprion* (42)] the neurocranium. As it stands, it is unclear how many times traits like holostyly and even durophagy evolved in the holocephalan total-group. Many extinct holocephalan taxa have only rarely been incorporated into phylogenetic analysis (20, 21), and untangling the evolution of the holocephalan body plan will ultimately rely on a clearer and more detailed picture of the phylogenetic relationships of these taxa than is currently available.

The evidence presented here for high-performance suction feeding in *Iniopera* expands known ecological niches exploited by holocephalans during the Carboniferous. *Iniopera*, which provides evidence for high-performance suction feeding in the holocephalan total-group, would have been able to exploit niches now all but monopolized by neopterygians, and in the Carboniferous only known to have been occupied by the stem-group elasmobranch *Tristychius* (4). Notably, and like *Tristychius* (4), this feeding strategy is undetectable with two-dimensional disparity studies (*SI Appendix*, Fig. S7). Whether iniopterygians as a whole radiated using high-performance suction feeding as a strategy is unclear; intercoracoid elements of the same shape are present in other Sibyrhinchidae from the Mecca Fauna (16), hinting that they employed this feeding strategy. In Iniopterygidae, the situation is unclear, although Grogan and Lund (17) identified complex oral cartilages that point toward a specialized feeding strategy. Clues as to their diet come from arthropods, conodont denticles, and plants reported in Mecca Fauna specimens (16), as well as a shrimp preserved inside a specimen from Bear Gulch (20), which would be consistent with their feeding in the water column. Although not preserved in *Iniopera* sp., all other iniopterygians have pharyngeal plates (16), comparable to pharyngeal dentitions in some teleosts, which were presumably important to feeding, but not mutually exclusive with suction feeding. Iniopterygians have no clear ecological analog among modern fishes, although they have been interpreted as being similar to eagle rays by Zangerl et al. (16) and appear to have a broad range of morphotypes including flattened forms (19) that suggests they may have radiated to a range of ecological niches. Marine vertebrate ecosystems in the Carboniferous were rewrought in the wake of the end-Devonian Hangenberg event, with recognizably modern groups—chondrichthyans, actinopterygians, and tetrapods—diversifying to fill extinction-emptied niches (43–45). Stem-group holocephalans were a major component of these radiating groups, diversifying into a far broader range of body shapes than the crown-group [e.g., *Belantsea* (46), *Chondrenchelys* (37)]. Many of these forms were durophagous (36, 47), but holocephalans also exploited feeding strategies untouched by the modern crown-group including a specialized clutching jaw action in the symmoriid *Ferromirum* (24), analogous to that of living snaggletooth sharks (48), and large symphyseal tooth whorls interpreted as an adaptation to hunting soft-bodied prey in eugeneodontids (42, 49). As *Iniopera* shows, these roles may also have included small-bodied fishes suction feeding in the water column, an ecological niche that later came to be dominated by neopterygians. Reinvestigation of the diverse array of Carboniferous stem-holocephalan forms seems likely to reveal further similar surprises.

## Materials and Methods

### Material Studied.

The *Iniopera* sp. material studied comprises two previously described specimens, as follows: KUVP 22060 and KUVP 158289 (26–29) (n.b. in these previous works KUVP 158289 is incorrectly referred to as "KUNHM 21894"). Both specimens are from the Upper Pennsylvanian (late Virgilian, 305 to 299 Ma) Haskell Formation, Kansas, US (26). The *Callorhinchus* is a late-stage embryo previously described by Pradel et al. (50) and Dearden et al. (30); all 3D models shown here are available via MorphoMuseum (51, 52). *Iniopera* is consistently recovered by phylogenetic analysis as a stem-group holocephalan (22–25), a position supported by morphological characters including a holostylic neurocranium, a jaw articulation on the extreme posterior end of the mandible, a subcranial branchial skeleton, the brain’s blood supply coming from the pseudobranchial rather than internal carotid arteries, a continuous series of basibranchial cartilages, and a subclavian artery passing through the scapulocoracoid symphysis (26–29).

### Software.

All reconstructions and analyses were carried out in Blender versions 2.9 to 3.1.2 (http://blender.org).

### Reconstruction.

The reconstructed *Iniopera* skeleton is a composite based on KUVP 22060 (skull, mandible, basihyal, basibranchial, and ceratohyal) and KUVP 158289 (shoulder girdle and ceratobranchial cartilages) (Fig. 1 and *SI Appendix*, Figs. S1 and S2). We imported models of all cartilages into Blender as separate .ply files. The components of the reconstruction are identical to those in figure 4 in ref. 29 with the exceptions that we corrected breaks in the scapulocoracoid and mandible that affected these elements’ shapes, using Blender to realign and remesh the elements (*SI Appendix*, Fig. S1 *E*–*L*). We reconstructed muscles based on the skeletal morphology of *Iniopera* using extant phylogenetic bracketing (53) with reference to the musculature in extant holocephalans and elasmobranchs (13, 30). Full justifications for muscle placement are given in *SI Appendix*, Fig. S2.

### Gape.

We estimated the gape of *Iniopera* by modifying the digital modeling approach of Lautenschlager (31) (Fig. 2 and *SI Appendix*, Fig. S4). The lower teeth are missing in *Iniopera* sp., but in other sibyrhynchids including *Iniopera*, teeth are present on the lower jaw (16). If *Iniopera* sp. displayed the same condition, the large “canine” whorls would be positioned to fit between the upper canine whorls (*SI Appendix*, Fig. S4*A*). For this reason, we estimated the maximum possible mandibular closure by copying the upper dentition onto the mandible and closing the jaw until teeth met (*SI Appendix*, Fig. S4*A*). We animated the mandible to rotate through 60 degrees using an armature spanning its articulations with the neurocranium (*SI Appendix*, Fig. S4*C*), starting at three plausible resting positions at 3, 6, and 9 degrees from the maximum mandibular closure. Next, 3D cylinders were used to model the mandibular adductor muscle and were placed at the anteriormost and posteriormost ends of the estimated origin and insertion sites on the closed jaws (*SI Appendix*, Fig. S4*B*). We parented these cylinders to single-bone armatures extending between the same two points, with their tail ends on the origin (*SI Appendix*, Fig. S4*D*). These armatures were given stretch-to bone constraints targeting their point of contact with the mandible, meaning that the armature and cylinder stretched parallel to the axis of the jaw’s rotation (*SI Appendix*, Fig. S4*D*). We modified a Python script from Lautenschlager (32) that was used to calculate the extension of the muscle cylinders and output the strain factor for each frame. Muscle cylinders were color-coded to correspond to within the optimal tension limit for mandibular adductor extension (green), within the maximum tension limit for mandibular adductor extension (yellow), and over the maximum tension limit (red). We captured renders of each frame to make figures.

### Ceratohyal Range of Motion.

We estimated the range of motion of the ceratohyal in *Iniopera* relative to the basihyal in Blender. The articulation between the basihyal and ceratohyal was interpreted as a hinge joint, with one degree of freedom, based on its previously described anatomy (29). This was represented with a cylinder, which was oriented with both articulation surfaces and the *z* axis of which was used as the joint axis (54). The ceratohyal was animated to rotate around this axis in 0.5-degree increments, and a Python script was written that detected overlap between the ceratohyal and basihyal meshes to determine the range of movement.

### Mechanical Advantage and Estimated Muscle Force.

We estimated the mechanical advantage of the jaw through the opening cycle using the 3D models of the neurocranium and mandible (Fig. 3 and *SI Appendix*, Fig. S5). The mandible was animated to rotate through to the estimated maximum possible extension of the mandibular adductor muscles over 160 frames. We added empties and parented them to models at the central positions of the mandibular adductor’s origin and insertion, at the point of rotation for the articulation of mandible and neurocranium, and at three points (anterior, middle, and posterior) along the biting surface of the mandible (*SI Appendix*, Fig. S5*A*). We wrote a Python script to output the coordinates of these empties in 3D space at each point in the animation and a script in R to calculate the vectors and lengths of levers involved in jaw closure. Three outlevers—the distance between the articulation and anterior, middle, and posterior points on the mandible—were used (*SI Appendix*, Fig. S5*B*). We took the inlever to be the distance between the articular joint and the center of the adductor fossa. The effective inlever (moment arm) was then calculated using the angle between the inlever and the summed vectors from this point to the of the two mandibular adductor origins at any given point. The outlever divided by the inlever was used to calculate mechanical advantage. The vector of the effective inlever was calculated as:

$$\mathrm{Inleve}r_{E}=\mathrm{Inleve}r_{A}\times \sin(\theta ),$$

where *θ* is the angle between the mandibular adductor and the Inlever_A_.

Mechanical advantage was calculated with:

$$\mathrm{Mechanical}\;\mathrm{advantage}=\frac{Inlever_{E}}{Outlever}.$$

Muscle force was calculated using:

$$F_{M}=\mathrm{Specific}\;\mathrm{tension}\times \mathrm{Area}\;of\;\mathrm{muscle}\;\mathrm{attachment}.$$

We interpret the mandibular adductor muscle in *Iniopera* as attaching to both the neurocranium and Meckelian cartilage without tendons as in living elasmobranchs (*SI Appendix*, SI text), and so took the area of muscle attachment as a proxy for the physiological cross-sectional area. We estimated this area using the surface area of the attachment surfaces on the 3D model, measured in Blender, and used the specific tension of elasmobranch skeletal muscle, namely, 28.9 Ncm^−2^ (55).

We then compared mechanical advantage and estimated muscle force with a dataset of posterior and anterior mechanical advantage and biting forces for extant chondrichthyans assembled by Motta and Huber (Table 6.2 in ref. 33) (Fig. 3 and *SI Appendix*, Fig. S5). Biting force was divided by body length, which in *Iniopera* was estimated using the length of the skull of our reconstruction and a full body reconstruction in Zangerl (18). We multiplied bite force in *Iniopera* by two to account for both sides of the mandibular adductor musculature having an effect, and so this is probably an overestimate. We also compared residuals from a linear regression of log10[bite force] against log10[body length] (*SI Appendix*, Fig. S5 *E–G*).

### Pharyngeal Expansion.

We estimated the expansion of the pharynx using the reconstructed models of the neurocranium, visceral, and pectoral skeleton to make an animated digital model of the skeleton’s motion during jaw and pectoral abduction (Fig. 4 and *SI Appendix*, Fig. S6). To simplify the model, we restricted motion to the sagittal plane. An armature was added along the midline linking the parts of the skeleton (neurocranium-suprascapular-scapulocoracoid-intercoracoid cartilage-basibranchial-basihyal) with a separate bone for each element (*SI Appendix*, Fig. S6 *A–C*). The joints between the neurocranium-suprascapular-scapulocoracoid were set to be stiff, so as to simulate their being embedded in muscle. We manipulated the armature by pulling posteriorly on the lower part of the scapulocoracoid via an additional bone added to the armature (*SI Appendix*, Fig. S6*C*). The basihyal was constrained to stay within a short distance of the mandible by adding an additional bone to the anterior end of the armature that was constrained to follow a curved rail set up behind the arc of mandibular movement. We set this cycle of movement up under two configurations, with the jaw set to open to the maximum gape based on the maximum tension limit of the reconstructed mandibular adductor muscles and the maximum of the optimal tension limit of the mandibular adductor muscles. In both cases, maximum pectoral abduction was estimated by pulling back the scapulocoracoid as far as possible at full gape. We also animated the ceratohyal to abduct through the same rotation as the maximum estimated by the range of motion analysis. We estimated pharyngeal expansion by creating a polyhedron mesh approximating the boundaries of the orobranchial cavity and filling half of the estimated pharyngeal volume (bisected in the sagittal plane), stretching from the front of the mouth to the level of the scapulocoracoids, which was manipulated to fill the bounds of the neurocranium and basibranchial skeleton (*SI Appendix*, Fig. S6*D*). Each vertex of this mesh was given its own vertex group and linked to an empty in the same position with a hook modifier. These empties were then parented to the surrounding *Iniopera* skeleton to allow the mesh to expand/contract posteriorly and laterally with the movement of the pharyngeal skeleton. We wrote a Python script that exported the change in volume of the pharynx into a text file and rendered each frame of the animation. A likely inaccuracy of our model is that its movements are synchronized, unlike in living fishes where there is an anterior-to-posterior wave of expansion (35, 56), although this should not impact our estimates of total volume change.

## Supplementary Material

Appendix 01 (PDF)

Movie S1. Animation of the gape opening in *Iniopera* with adductor muscles modelled as cylinders, following method of Lautenschlager *(29)*, starting at resting position of 3 degrees.

Movie S2. Animation of the gape opening in *Iniopera* with adductor muscles modelled as cylinders, following method of Lautenschlager *(29)*, starting at resting position of 6 degrees.

Movie S3. Animation of the gape opening in *Iniopera* with adductor muscles modelled as cylinders, following method of Lautenschlager *(29)*, starting at resting position of 9 degrees.

Movie S4. Animation of reconstructed pharyngeal expansion in *Iniopera*, with the mouth opening to the optimal extension of mandibular adductor muscles.

Movie S5. Animation of reconstructed pharyngeal expansion in *Iniopera*, with the mouth opening to the maximum extension of mandibular adductor muscles.

## Data Availability

All Python scripts, R scripts, and Blender files are available in the GitHub repository at the following link: https://github.com/rpdearden/Iniopera_suction. The original tomographic data for both specimens that the analysis is based on are available at Morphosource: specimen KUVP 22060 at http://n2t.net/ark:/87602/m4/478221 (57) and specimen KUVP 158289 at https://github.com/rpdearden/Iniopera_suction (58). All 3D models are also available at Morphomuseum (51, 52).
