# Supplementary material for: Evidence for high-performance suction feeding in the Pennsylvanian stem-group holocephalan Iniopera
Source: Proc Natl Acad Sci U S A. 2023 Jan 17;120(4):e2207854119. doi: 10.1073/pnas.2207854119 (PMC9942859; doi:10.1073/pnas.2207854119)
Supplement: Supplementary file 1 — Appendix 01 (PDF) [file pnas.2207854119.sapp.pdf]

PNAS
www.pnas.org

Supplementary Information for

Evidence for high-performance suction feeding in the Pennsylvanian stem-group holocephalan *Iniopera*

Richard P. Dearden, Anthony Herrel, Alan Pradel

Richard P. Dearden Email:
richard.dearden@bham.ac.uk

This PDF file includes:

Supplementary text
Figures S1 to S7
Legends for Movies S1 to S5
SI References

Other supplementary materials for this manuscript include the following:

Movies S1 to S5

Supplementary Information Text

Details of skeletal 3D model

We built a model to approximate the range of movement of the pharyngeal and pectoral skeleton of *Iniopera*, using the 3D models described in the Methods (Figs 1, S1-S2). For detailed anatomical descriptions of components see [1–4]. The models were those used for the reconstruction of Pradel *et al.*[4] but with the following changes in positioning made compared to their reconstruction. Two specimens were used, KUVP 22060 and KUVP 158289. Of these, KUVP 158289 has in previous publications been referred to as “21894”[1–4]; the specimen number has subsequently been revised due to a specimen number duplication in the KUVP collections.

The neurocranium, mandible, and upper tooth whorls are from KUNHM 22060[1] (figs 1-13, 31-32). Lower tooth whorls are missing from the specimen but are assumed to have had a similar morphology based on the mandible’s shape and flattened *Iniopera* specimens[5]. Upper tooth whorls positions were estimated based on comparison to other *Iniopera* specimens[1,5]. A break in the mandible that affected its alignment was fixed by cutting along the line of the break, rotating the two parts to fit and reattaching them in Blender; it was then remeshed (Fig. S1j-g).

The mandible was fitted into articulation with the neurocranium; its resting position was approximated by copying models of the upper tooth whorls onto the lower jaw and closing the jaw until they made contact (Fig. S3a). The neurocranium and mandible were both remeshed in Blender to provide smaller more manageable models.

The intercoracoid, scapulocoracoid, suprascapular elements, and fin elements are from KUNHM 158289[2] (figs 1+2). Paired elements (scapulocoracoid, fin skeleton, and suprascapular) are from the right-hand side of the fossil and are mirrored across the centre of our model. Like with the mandible, a break in the scapulocoracoid was that affected its alignment was fixed by cutting along the line of the break, rotating the two parts to fit and reattaching them in Blender; it was then remeshed (Fig. S1k-n).

Additional visceral cartilages are taken from both KUNHM 22060 — basihyal, basibranchial, ceratohyal [4] (fig. 1)— and KUNHM 158289— ceratobranchials [4] (fig. 2). Ceratohyals were articulated with corresponding facets on the basibranchial, their exact angle and position is unknown as is the presence of an epihyal/hyomandibula (Figs 1, S1). Unlike extant holocephalans the ceratohyals seem to have lain out of sequence of the branchial arches, and it is unknown whether the epihyal articulated with the braincase, although the lack of an obvious articulation surface makes it unlikely[1]. Ceratobranchials were used to inform the approximate shape and size of the orobranchial cavity (Figs 4, S6) but their exact placement is conjectural, and the presence or absence of hypo-, epi-, and pharyngobranchials is unknown[4].

The exact placement of neurocranial, visceral, and pectoral elements relative to one another is necessarily subjective, but preserved positions and the articulations between elements were used as a guide to their relative positions.

Muscle placement justifications

Detailed justification for reconstructions of muscles related to high performance suction feeding in *Iniopera* follows. Muscles in *Iniopera* are reconstructed using the extant phylogenetic bracket[6] with particular reference to holocephalans and elasmobranchs.

<u>*M. adductor mandibulae.*</u>

This muscle is present in both holocephalans and elasmobranchs, and is innervated by the maxillary branch of the trigeminal (V) nerve[7,8]. In holocephalans the muscle has a broad preorbital origin on the neurocranium, and inserts on a sheet of connective tissue slung around the bottom of Meckel’s cartilage (Figs 1b, S2f)[8,9]. In elasmobranchs the muscle has its origin on the palatoquadrate and inserts directly on the posterior part of the lateral face of Meckel’s cartilage[9,10].

Iniopera has a holostylic neurocranium and so this muscle must have had its origin on the neurocranium as in living holocephalans. *Iniopera* lacks the high-walled lamina orbitonasalis present in crown-group holocephalans but there is a large preorbital fossa, ventral to the nasal capsules, which we consider to a probable position for the mandibular adductor's antorbital origin (Figs 1a, S1d, S2a). A foramen in the wall of this fossa is interpreted by Pradel *et al.*[1] as providing passage for the maxillary branch of the trigeminal nerve[1] (fig. 6B, *fVmx*), further supporting this interpretation. There also seems likely to have been an insertion in the bottom of the orbit, as suggested by Dearden *et al.*[9]. We have reconstructed the muscle with these two origins, analogous to the suborbital and antorbital mandibular adductor muscles in living holocephalans[9]. Pradel *et al* [1] suggest instead that the origin of the mandibular adductor was on the ventral part of the postorbital wall: this seems unlikely as the muscle would have occluded the orbit.

The insertion of the muscle was on the lateral side of Meckel's cartilage (Figs 1[a], S1d, S2a,c). Notably the cartilage is demarcated by a ventral rim meaning that the muscle must have inserted on the cartilage itself as in crown-group elasmobranchs, rather than on a ventral sling as in crown-group holocephalans[8,9,11].

Mm. anguli oris, M. labialis anterior, M. intermandibularis

In crown-group holocephalans, these muscles insert on the labial cartilages. The *m. anguli oris anterior* and the *m. anguli oris posterior* have preorbital origins and attach on the labial cartilages, the *m. labialis anterior* muscle connects certain labial cartilages, and the *m. intermandibularis* comprises two sections which link certain labial cartilages to the Meckelian cartilage; all are innervated by the trigeminal (V) nerve[8,9]. None of these muscles are present in crown-group elasmobranchs[9].

In *Iniopera* there is no evidence for labial cartilages having been present[1] and no evidence for the presence of these muscles.

M. superficialis

This superficial muscle, innervated by the trigeminal (V) nerve, has only been reported in *Callorhinchus* among crown-holocephalans[8,9] and its presence in *Iniopera* is unknowable from the preserved remains.

M. mandibulohyoideus

In crown-group holocephalans the *m. mandibulohyoideus* has an origin on the posterior of the Meckelian symphysis and inserts on the ventral angle of the ceratohyal (Fig. 1f, S2g) [8,9,12]. It is innervated by the facial (VII) nerve as well as the glossopharyngeal (IX) nerve in *Hydrolagus* [8,9,12]. This muscle is absent in elasmobranchs and is likely apomorphic for total-group holocephalans [12].

Iniopera lacks the insertion area on the postero-ventral part of the mandibular symphysis onto which this muscle has its origin in *Callorhinchus* (Fig. S2h)[9]. However, the ceratohyal does possess a large ventral angle similar to that which the muscle inserts on in living holocephalans (Fig. 1a, S1b) and which is absent in elasmobranch ceratohyals[4,9]. This muscle is the only candidate from the muscles of living chondrichthyans for attachment in this position, and seems likely to have been present in *Iniopera* (Fig. 1a,e). We consider it likely that it was present but it seems unlikely that it would have attached to the mandibular symphysis as in living holocephalans: its path is obstructed by the basihyal, instead it may have attached on the medial face of the Meckelian cartilage.

M. epaxialis

In living holocephalans the epaxial muscles attach over a large area of the dorsal surface of the neurocranium, on either side of the endolymphatic foramen (Figs 1b, S2f). In elasmobranchs these muscles attach in a similar position [8,9]. They are innervated by spinal nerves [7].

In *Iniopera* fossae are present on either side of the endolymphatic foramen and occipital crest which where the epaxial muscles would have inserted (Figs 1a, S1e,f, S2a-d)[1] (fig. 6, *pf*). Compared to *Callorhinchus* the insertion area for these muscles on the neurocranium is limited [9]. Foraminae are present in the occipital region of *Iniopera* which may have carried spino-occipital

nerves to innervate the epaxial musculature[1](fig. 21, *so*, *bso*). The lateral extent of the epaxial muscles would have been limited by the shoulder girdle.

M. protractor dorsalis pectoralis

In crown-group holocephalans the *m. protractor dorsalis pectoralis* has its origin posterior to the orbit, ventral to the lateral otic ridge and inserts on the anterior edge and medial side of the upper scapular process[9] where it meets the *m. retractor dorsalis pectoralis* (Figs 1a, s!b, S2a,c,d,e). The *m. protractor dorsalis pectoralis* is probably a trunk muscle, innervated by the nerves IX and X[13,14]. This muscle is absent in elasmobranchs.

Iniopera possesses extremely large postorbital fossae on either side of the occiput (Fig. S2d) [1](fig. 7, *ld*). Pradel *et al* suggest that these probably accommodated the "jaw and visceral musculature (e.g. the *cucullaris profundus, subspinalis,* and *levator hyoideus* muscles) and gills. This is probably the case given their position relative to the branchial skeleton in our construction (see below). However, these muscles in living holocephalans are collectively fairly small. Their size and the large fossae that face them on the scapulocoracoid (Fig. S2e) suggest that they mainly served as origins for muscles linking the neurocranium and shoulder girdle. The closest analogue of this in living chondrichthyans is the *m. protractor dorsalis pectoralis* and it seems likely that a similar muscle joined the scapulocoracoid and neurocranium in *Iniopera*. In all figures we have termed this muscle *m. protractor dorsalis pectoralis*, but the exact homology of the muscle in this position is unclear.

M. retractor dorsalis pectoralis

In living holocephalans the *m. retractor dorsalis pectoralis* has its origin in the trunk musculature and inserts on the medial face of the upper scapulocoracoid (Figs 1b, S2g,h,i)[9]. As with the *m. protractor dorsalis pectoralis* it is probably a trunk muscle[13,14] and is absent in elasmobranchs. It is innervated by the spinal nerves.

The large areas on the posterior face of the scapulocoracoid of *Iniopera* suggest a large area for insertion of posteriorly oriented musculature (Figs 1a, S1f,e, S2e), which may have been homologous to the *m. retractor dorsalis pectoralis*.

M. retractor mesio-ventralis pectoralis

In living holocephalans *m. retractor mesio-ventralis pectoralis* is a part of the trunk musculature that inserts on the ventral part of the scapulocoracoid (Figs 1b,f, S2g)[8,9]. In elasmobranchs hypaxial muscles insert posteriorly on the scapulocoracoid [15]. It is innervated by the spinal nerves.

Iniopera has large, posteriorly facing surfaces on the rear of the scapulocoracoids, which likely served as insertions surfaces for some kind of ventral musculature (Fig. 1a,e, S1f, S2b). Whether this was a distinct muscle or not is unclear, but for ease of comparison with *Callorhinchus* we have labelled these as *m. retractor mesio-ventralis pectoralis* in our figures.

M. cucullaris superficialis

In living holocephalans the *m. cucullaris superficialis* overlies the *m. protractor dorsalis pectoralis* with an origin on the postorbital crest (Figs 1b, S2f,i)[8,9], and inserts on the lateral face of the scapulocoracoid. It is innervated by nerves IX and X. It is absent in elasmobranchs.

In *Iniopera* the postorbital wall [1](fig. 6B, *pow*) is divided into an upper and a lower part by a ridge [1] (fig. 6B, *imz*). Pradel *et al.* [1] suggest that the upper part may have been the origin of a *m. cucullaris superficialis*: this seems likely (Figs 1a, S1d,e, S2a). If so it would have inserted on the lateral part of the scapulocoracoid (Figs 1a, S2a,e). It seems likely that a separate *m. cucullaris profundus* and *m. cucullaris superficialis* like living holocephalans were present due to the subcranial pharynx and this candidate origin site.

M. cucullaris profundus/m. subspinalis

In living holocephalans the *m. cucullaris profundus* has its origin on the neurocranial floor and inserts on the posterior pharyngobranchial complex (Fig. S2h) and is innervated by nerves IX and X[8,9]. The *m. subspinalis* has its origin medial to this and inserts on the first two pharyngobranchials (Fig. S2h) and is innervated by spinal nerves and cranial nerve X. In elasmobranchs both of these muscles are also present. The *m. cucullaris profundus* has its origin posteriorly to the neurocranium and inserts on the posterior pharyngobranchial complex and the scapulocoracoid. The *m. subspinalis* has its origin on the back of the neurocranium and inserts on pharyngobranchial I.

These muscles are present in living elasmobranchs and holocephalans and so were likely present in *Iniopera*. For the reasons outlined above, we consider it likely that *Iniopera* had a separate *m. cucullaris profundus* and *m. cucullaris superficialis* like living holocephalans. However, no muscle attachments are visible, and the pharyngobranchials are not preserved. As previously suggested[1] these muscles presumably both inserted somewhere in the large postorbital fossae with the *protractor dorsalis pectoralis* equivalent as the only possible insertion point place dorsal to the branchial skeleton (see above).

<u>M. levator hyoideus</u>

In living holocephalans the *m. levator hyoideus* has its origin on the basicranium and inserts on the epihyal [8,9]. It is innervated by cranial nerve VII. In elasmobranchs this muscle is present but incorporated into the dorsal hyoid constrictor muscle to varying degrees [10].

Posterior to the orbits in *Iniopera* there is a small scar (fig. 10, *mi of* [1]) which could feasibly have been the origin point for this muscle. Another possibility is that it as the articulation of the hyoid arch, although the hyomandibula and whether it articulated with the neurocranium is unknown in *Iniopera* and

<u>M. coracomandibularis</u>

In living holocephalans the *m. coracomandibularis* inserts on the posterior edge of the mandible and has its origin on the coracoid and via two arms, on the bases of the scapular processes (Figs 1b, 1f, S2f-i)[8,9]. It is innervated by the spinal nerves. It is also present in elasmobranchs in which it is a proportionally thinner muscle which also extends from the mandible to the coracoid.

In *Iniopera* this muscle presumably inserted on or along the bottom of the mandible, although it lacks the attachment area present in living holocephalans (Figs 1a,e, S1d, S2a,d,e). *Iniopera* also lacks the fused coracoid of living holocephalans. The origin of the *m. coracomandibularis* may have been on some combination of the bottom of the intercoracoid element, and may also have lain on the intercoracoid element, where there is a plausible attachment surface.

<u>M. coracohyoideus</u>

In living holocephalans the origin of the *m. coracohyoideus* is on top of the *m. coracomandibularis* (Figs 1b,f, S2h). It then inserts on the posterior face of the basihyal[8,9]. It is innervated by the spinal nerves. It is also present in elasmobranchs, with the same insertion and origin points.

In *Iniopera* they probably inserted on the much larger attachment surface along the ventral posterior edge of the basihyal (Figs 1a,e, S1d, S2d,e). The origin was possibly on the anterior face of the scapulocoracoid, or on the dorsal side of the *m. coracomandibularis*. Their relative size appears likely to have been much larger in *Iniopera* than in living holocephalans and probably played a more major role relative to the smaller *m. coracomandibularis*.

<u>Mm. coracobranchiales</u>

In living holocephalans the *mm. coracobranchiales* have their origins along the medial margin of the ventral scapular shaft and insert between the hypobranchials (Fig. S2f,i)[8,9]. It is innervated by the spinal nerves. In elasmobranchs they have their origin along the edges of the coracoid and insert on the hypobranchials.

It seems likely in Iniopera the *mm. coracobranchiales* had their origins along the side of the lower scapulocoracoid (Fig. S2a,e), as the broad basibranchial covered the intercoracoid. Insertions are not known, but presumably lay on the hypobranchials.

<u>*M. constrictor operculi dorsalis* and *ventralis*</u>

Living holocephalans have a single *m. constrictor operculi dorsalis* and *ventralis* (Fig. S2h)[8,9], both of which are innervated by cranial nerve VII. The *m. constrictor operculi dorsalis* has its origin at the base of the scapular process and on the notochord, and inserts along the rim of the operculum as well as along the bottom edge of the orbit. The *m. constrictor operculi ventralis* has its origin on the opercular cover and inserts ventrally. Elasmobranchs instead have a *m. constrictor hyoideus dorsalis*, which has its origin across the otic part of the neurocranium and epaxials and inserts on its ventral counterpart and the hyomandibula, and a *m. constrictor hyoideus ventralis*, which has its origin along the median aponeurosis and inserts on its dorsal counterpart[9].

No mineralised opercular cartilage is present in *Iniopera*, but one may have been carried by the large ceratohyal[4]; the subcranial pharynx suggests that it probably had a single branchial opening like living holocephalans and that these muscles were more like those of living holocephalans than elasmobranchs. It seems likely that the *m. constrictor operculi dorsalis* had a suborbital origin on the coracoid and on the lamina ventral to the ridge in the postorbital wall, identified by Pradel *et al.*[1] as a possible mandibular adductor site.

Details of the 3D pharyngeal expansion animation

The model was animated in Blender using a single armature with a different bone for each component, placed down the centre of the model (Fig. S6). Joints between bones were defined as below and were translated in the X axis to provide the locations for each armature joint. 3D models of the individual skeletal elements were linked to armature bones using the “child of” object constraint. Paired elements (e.g. the scapulocoracoid) were mirrored around a central point. A posterior bone was added to the armature which was linked to an empty using the “inverse kinematics” bone constraint. This empty then moved posteriorly to simulate the contraction of the ventral trunk muscles. An anterior bone was added to the armature to simulate the basihyal being pulled posteroventrally. This was linked to an empty using the “inverse kinematics” bone constraint. The empty was then moved postero-ventrally along a curve.

To approximate pharyngeal expansion a polyhedron mesh was created which was shaped to fit one half of the interior of the buccal cavity at a closed position (Fig. S6d). Each vertex of this mesh was assigned to a different vertex group, each of which was then “attached” with a hook modifier to an empty in the same position as each vertex. These empties were then parented to parts of the skeleton of *Iniopera* so that the polyhedron moved with the animated model.

<u>Neurocranium-suprascapular joint</u>

There is no joint between the suprascapular and the neurocranium, but based on its preserved position it may articulate with a process on the back of the braincase [2] and may have been attached by a ligament (Figs S1,S6). This places the element at the lateral edge of the epaxial muscles ’attachment to the neurocranium, and by comparison with living holocephalan musculature it seems likely that the element was embedded between the epaxial musculature and the musculature linking the braincase to the scapulocoracoid (see below), perhaps to provide firm dorsal anchorage for the shoulder girdles movements. In our model we have enabled rotation between the neurocranium and suprascapular but have stiffened the joint quite substantially to simulate its likely embedding in muscle.

<u>Suprascapular-scapulocoracoid</u>

Again, there is no obvious joint between the suprascapular and scapulocoracoid, but based on its preserved position it seems to have overlain the top of the scapulocoracoid (Figs S1, S6)[2]. Again,

it may have been attached via a ligament. In our model we have enabled rotation between the suprascapular and the top of the scapulocoracoid but have stiffened the joint quite substantially to simulate its likely embedding in muscle.

Scapulocoracoid-Intercoracoid

The intercoracoid and scapulocoracoids have a joint formed by paired rounded bulges on the posterior edge of the intercoracoid that fit into sockets in the upper face of the scapulocoracoids (Figs S1, S6)[2]. It seems likely these would have permitted quite free movement both dorso-ventrally and laterally. In our model this is represented by free rotation and no stiffness modifier.

Intercoracoid-Basibranchial

A laterally elongated facet on the front of the intercoracoid meets a rounded ridge on the ventro-posterior face of the basibranchial (Figs S1,S6). This seems likely to have permitted some limited rotation around the transverse axis. This is represented in our model by a stiff joint that can rotate around this axis

Basibranchial-basihyal

The basibranchial has two anterior processes which match facets on the posterior side of the basihyal (Figs S1, S6). This would have permitted some rotation around the transverse axis. This is represented in our model by a stiff joint that can rotate around this axis

Basihyal-Mandible

There is no articulation between the basihyal and the mandible (Figs S1,S6). However, the shape of the basihyal closely fits the shape of the mandible and its large size suggests that it probably acted somewhat like the ‘tongue ’formed by the basihyal in living elasmobranchs and would have had limited movement relative to the mandible being embedded in the pharyngeal musculature. This is represented in our model by its movement following a curve posterior to the Meckelian cartilage.

Ceratohyal

The ceratohyal was not included in the armature described above, but was animated separately. The resting position of the ceratohyal relative to the basihyal was estimated as part of the range of motion analysis (see main methods, Figs 2, S3). It was copied into this model in the same position and parented to the basihyal. It was then abducted around the same axis as was used for the range of motion analysis, ending at the maximum possible angle of abduction as was calculated by the that analysis.

Supplementary figures

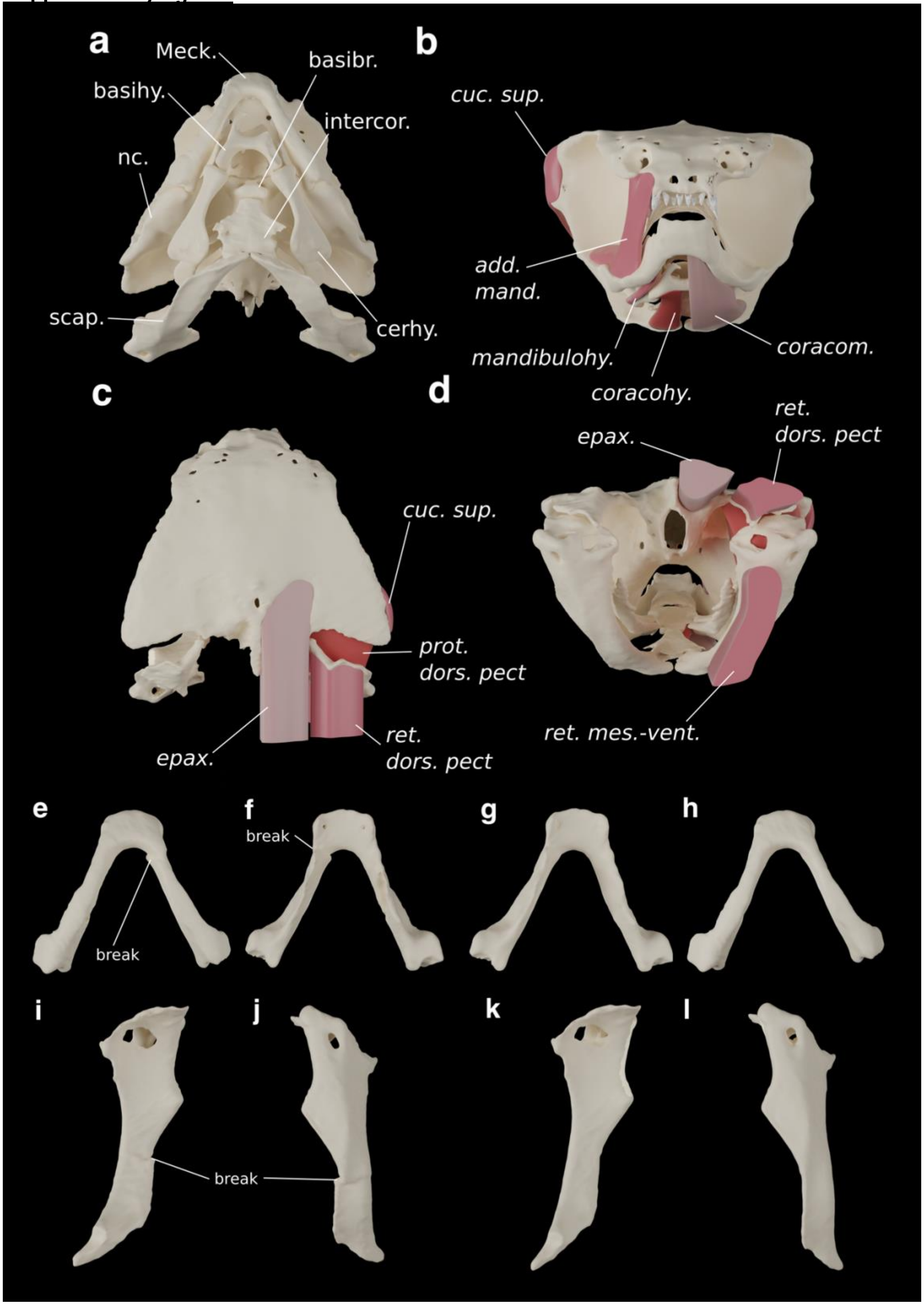

Figure S1. Additional images of *Iniopera* muscle reconstruction from Figure 1. a, reconstructed cranial skeleton of *Iniopera* in ventral view. b-c reconstructed musculature and cranial skeleton of *Iniopera* shown in anterior (b), dorsal (c), and posterior (d) views. e-f, original Meckelian cartilage with break shown in ventral (e) and dorsal (f) view. g-h, Meckelian cartilage with break corrected shown in (g) dorsal and (h) ventral view. i-j, original scapulocoracoid with break shown in (i) anterior and (j) posterior view. k-l, scapulocoracoid with break corrected shown in (k) anterior, and (k) posterior view. Abbreviations: *add. mand.*, *adductor mandibularis* muscle; basihy., basihyal; basipt, basipterygium; cerbr, ceratobranchials; cerhy, ceratohyal; *coracohy.*, *coracohyoideus* muscle; coracom., *coracomandibularis* muscle; *cuc sup*. *cuccullaris superficialis* muscle; *epax*., *epaxialis* muscles; intercor., intercoracoid element; *mandibulohy., mandibulohyoideus* muscle; Meck., Meckel's cartilage; nc., neurocranium; *prot. dors. pect.*, *protractor dorsalis pectoralis* muscle; *ret*. *dors*.-*pect*., *retractor dorsalis pectoralis* muscle; rad, fin radial; *ret. mes.-vent*., *retractor mesio-ventralis* muscle ; scap., scapulocoracoid ; sup. suprascapular element.

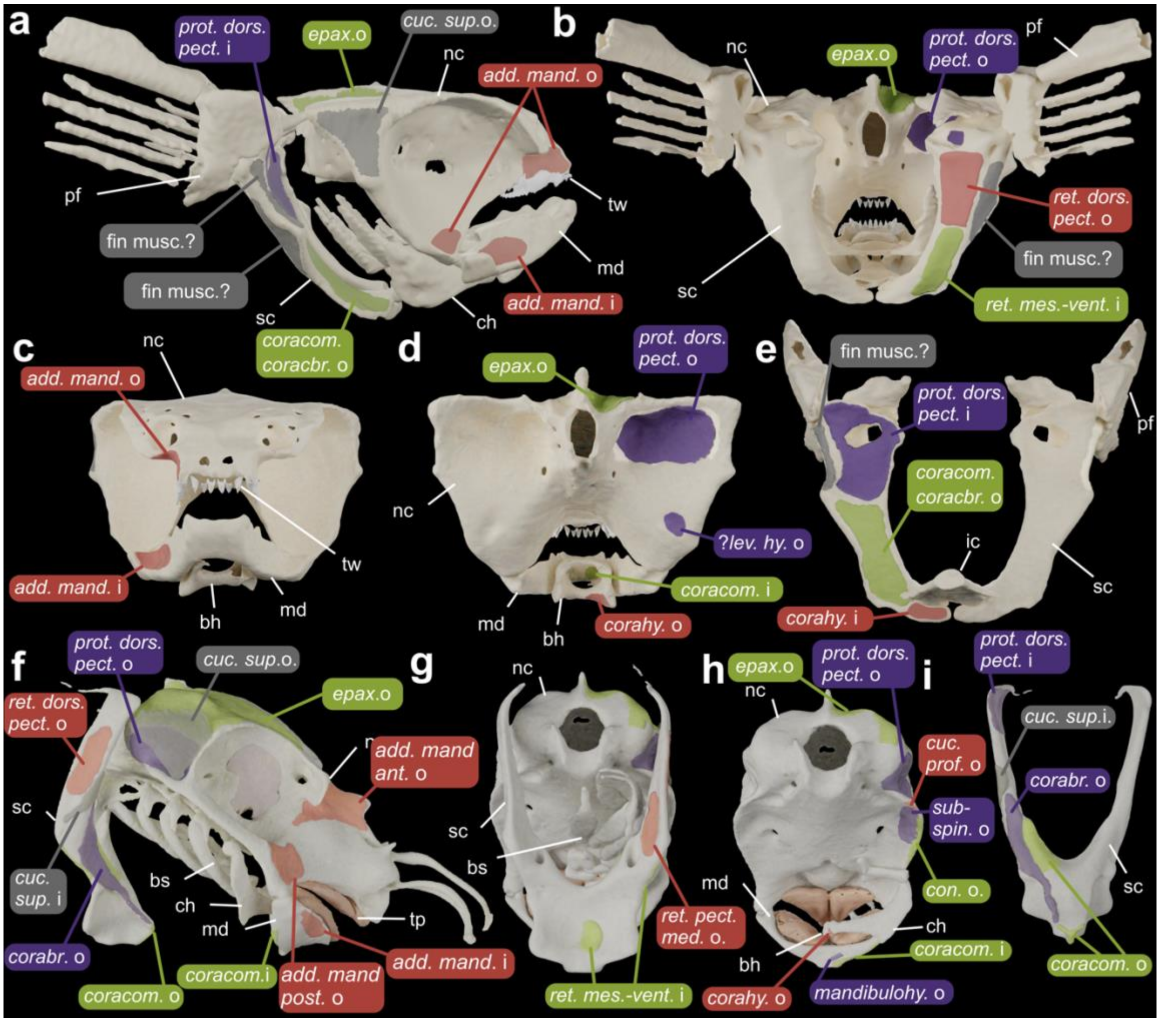

Figure S2. Reconstruction of cranial muscle attachments relevant to suction feeding in *Iniopera*, compared to *Callorhinchus*. a-e, reconstructed cranial, visceral, and pectoral skeleton of *Iniopera* in (a) right lateral, (b) posterior view, (c) skull, mandible and basihyal of *Iniopera* in anterior, and (d) posterior view and (e) pectoral girdle of *Iniopera* in anterior view. f-i skull, visceral, and pectoral skeleton of *Callorhinchus* in (f) right lateral (g) posterior view, (h) neurocranium in posterior view and (i) shoulder girdle in anterior view. Skeletal abbreviations (black): bh, basihyal; bs, branchial skeleton; ch, ceratohyal; ic, intercoracoid; md, mandible; nc, neurocranium; pf, pectoral fin; sc, scapulocoracoid; tp, tooth plates; tw, tooth whorls. Muscle Abbreviations (colour): CB, coracobranchiales; CH, coracohyoideus; CM coracomandibularis; CP, cucullaris profundus; CS, cucullaris superficialis; Ep, epaxial muscles; I, Insertion site; MA, mandibular adductor; MAa, mandibular adductor anterior; MAp, mandibular adductor posterior; O, origin site; Op, constrictor operculum dorsalis; PAb, pectoral adductor; PAd, pectoral adductor; PDP, protractor dorsalis pectoralis; RMVP, retractor mesio-ventralis pectoralis.

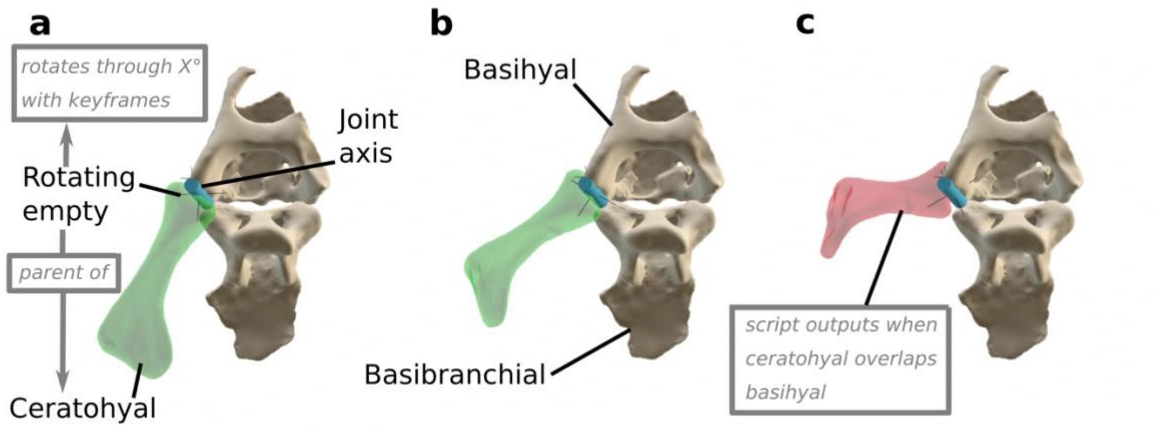

Figure S3. Blender setup for hyoid range of motion analysis for *Iniopera*. a-c, basihyal, ceratohyal, and basibranchial in ventral view with basihyal in (a) resting position, (b) maximum abduction before overlap with basihyal, and (c) beyond this position. Grey boxes represent operations, constraints, and modifiers in Blender.

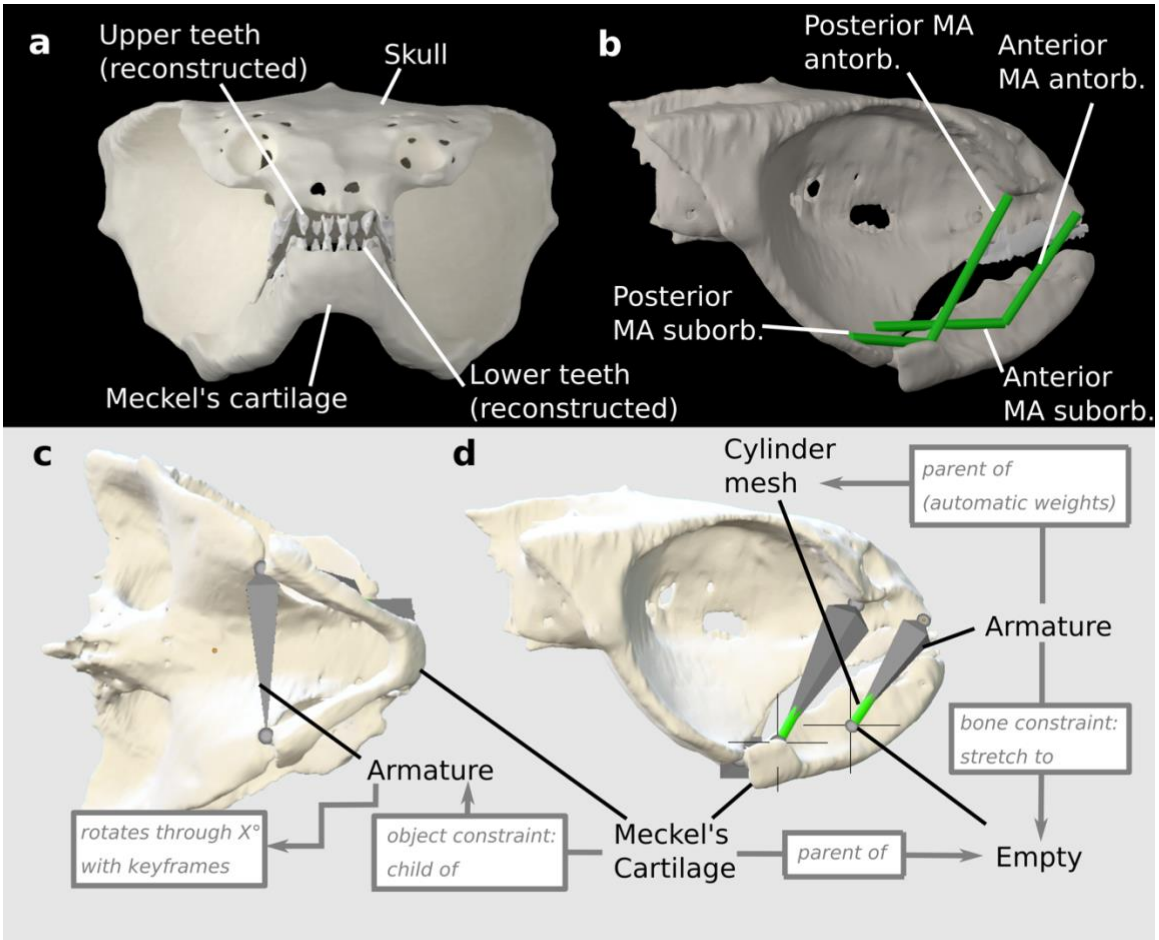

Figure S4. Blender setup for gape analysis in *Iniopera*. a, anterior view of head skeleton showing 0° gape, with near occlusion of reconstructed upper dentition and estimated lower dentition. b, right lateral view of skull showing positions of cylinder mandibular adductor models. c and d skull with explanation of blender operations using the antorbital mandibular adductor as an example (setup for suborbital is identical) (c) in ventral view showing Meckel-rotation armature and (d) in right lateral view showing the gape armatures. Grey boxes represent operations, constraints, and modifiers in Blender.

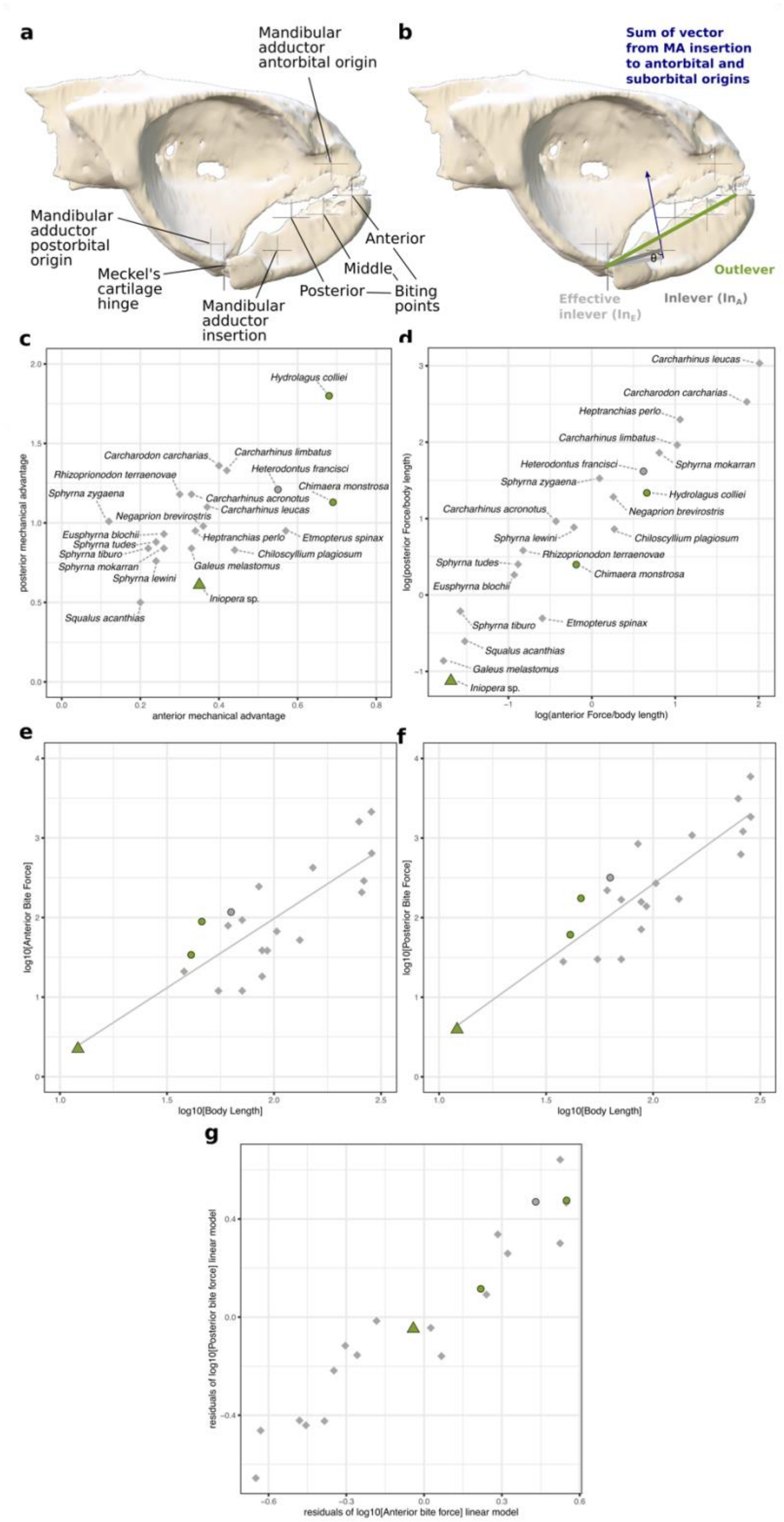

Figure S5. Additional information on mechanical advantage and force estimate of the jaws of *Iniopera*. a, positions of different measurement points on the head skeleton. b, diagram showing different levers, with jaws in a closed position. c and d graphs from Fig. 3b,c with taxa labelled. e-g, results of linear models of the logged anterior (e) and posterior (f) bite force compared to logged body length, with the residuals of both compared (g). Symbol key identical to Fig. 3.

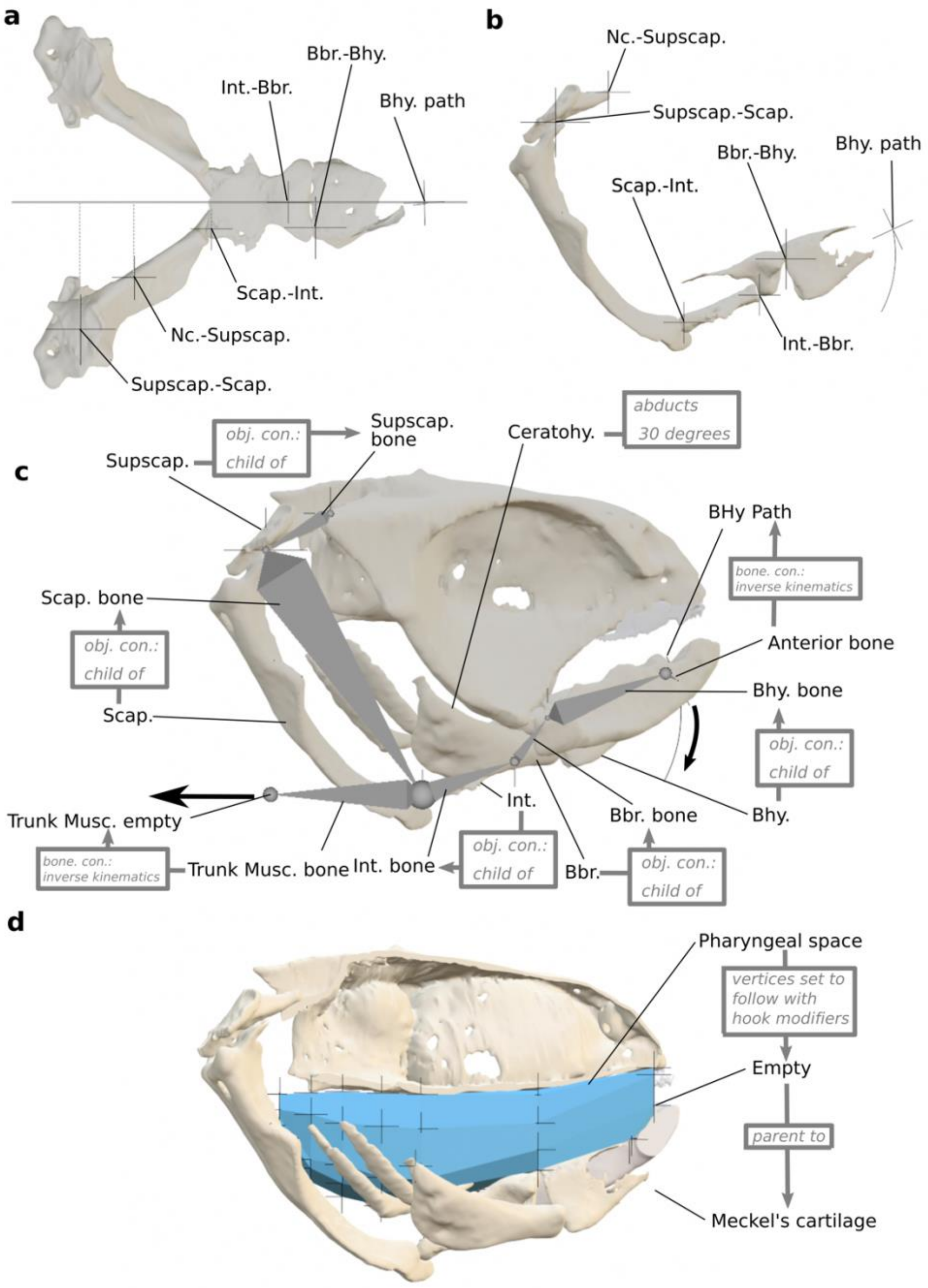

Figure S6. Blender setup for gape analysis in *Iniopera*. a,b Linked animated bones in dorsal (a) and right lateral (b) views. Crosses represent positions of joints, centre line in a is the midline along which the armature was placed, with joints translated medially along dotted lines. c. armature used to animate head in right lateral view, overlying skeleton, with explanation of blender operations. d. shape used to estimate changes in pharyngeal volume shown in right lateral view, with head skeleton split down midline. The relationship of one vertex of the pharyngeal polyhedron to the moving model is shown as an example. Other vertices tracked the movement of the shoulder girdle, hyoid, and basibranchial skeleton. Grey boxes represent operations, constraints, and modifiers in Blender.

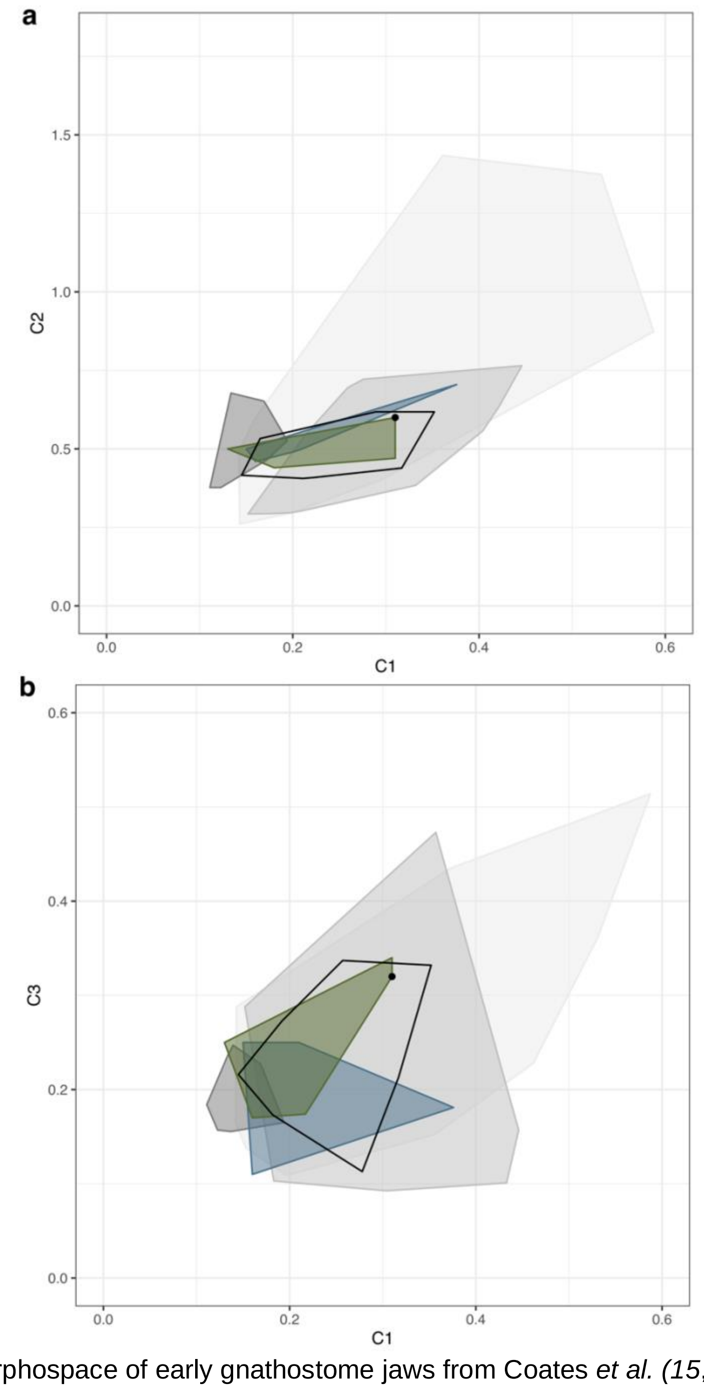

Fig. S7. Morphospace of early gnathostome jaws from Coates *et al. (15*, fig. S8) with *Iniopera* highlighted. Dataset originally modified from Anderson *et al.(16)* with additions by Coates *et al. (4)* a, measurement C1 plotted against C2. b, measurement C1 plotted against C3. Colour scheme: *Iniopera*, black dot; light grey, sarcopterygians; mid-grey, placoderms; dark grey, actinopterygians; blue, elasmobranchs; green, holocephalans; black line, acanthodians.

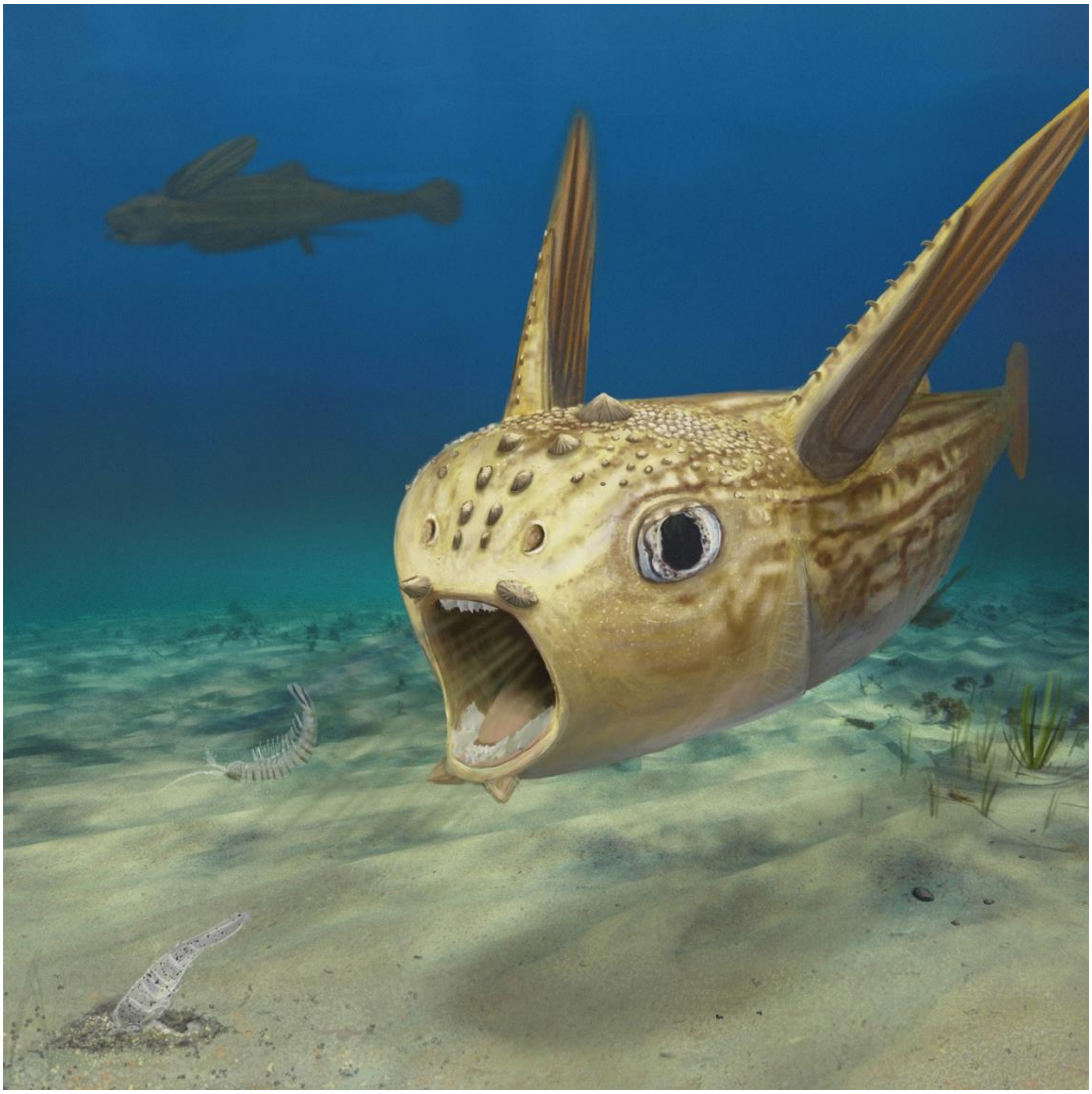

Fig. S8. An artists interpretation of *Iniopera* suction feeding. By Sophie Fernandez (MNHN).

Movie S1. Animation of the gape opening in *Iniopera* with adductor muscles modelled as cylinders, following method of Lautenschlager *(29)*, starting at resting position of 3 degrees.

Movie S2. Animation of the gape opening in *Iniopera* with adductor muscles modelled as cylinders, following method of Lautenschlager *(29)*, starting at resting position of 6 degrees.

Movie S3. Animation of the gape opening in *Iniopera* with adductor muscles modelled as cylinders, following method of Lautenschlager *(29)*, starting at resting position of 9 degrees.

Movie S4. Animation of reconstructed pharyngeal expansion in *Iniopera,* with the mouth opening to the optimal extension of mandibular adductor muscles.

Movie S5. Animation of reconstructed pharyngeal expansion in *Iniopera,* with the mouth opening to the maximum extension of mandibular adductor muscles.

SI References

1. A. Pradel, Skull and brain anatomy of Late Carboniferous Sibyrhynchidae Skull and brain anatomy of Late Carboniferous Sibyrhynchidae (Chondrichthyes, Iniopterygia) from Kansas and Oklahoma (USA). *Geodiversitas* **32**, 595–661 (2010).
2. A. Pradel, P. Tafforeau, & P. Janvier, Study of the pectoral girdle and fins of the Late Carboniferous sibyrhynchid iniopterygians (Vertebrata, Chondrichthyes, Iniopterygia) from Kansas and Oklahoma (USA) by means of microtomography, with comments on iniopterygian relationships. *Comptes Rendus Palevol* **9**, 377–387 (2010).
3. A. Pradel, *et al.* Skull and brain of a 300-million-year-old chimaeroid fish revealed by synchrotron holotomography. *Proceedings of the National Academy of Sciences of the United States of America* **106**, 5224–5228 (2009).
4. A. Pradel, R. P. Dearden, A. Cuckovic, R. Mansuit, & P. Janvier, The visceral skeleton and its relation to the head circulatory system of both a fossil, the Carboniferous *Iniopera*, and a modern, *Callorhinchus milii* holocephalan (Chondrichthyes). in *Ancient Fishes and their living relatives: a tribute to John G Maisey* Ed. Pradel A, Denton, JSS, and Janvier P (2021)
5. R. Zangerl, & G. Case, Iniopterygia, a new order of chondrichthyan fishes from the Pennsylvanian of North America. *Fieldiana* **6**, (1973).
6. L. M. Witmer, The extant phylogenetic bracket and the importance of reconstructing soft tissues in fossils. in *Functional morphology in vertebrate paleontology* 16 (Cambridge University Press, 1995).
7. F. H. Edgeworth, *The cranial muscles of vertebrates*. (Cambridge University Press, 1935).
8. D. A. Didier Phylogenetic Systematics of Extant Chimaeroid Fishes (Holocephali, Chimaeroidei). *American Museum Novitates* (1995).
9. R. P. Dearden, *et al.* The morphology and evolution of chondrichthyan cranial muscles: a digital dissection of the elephantfish *Callorhinchus milii* and the catshark *Scyliorhinus canicula*. *Journal of Anatomy* (2021).
10. M. C. Soares, & M. R. D. Carvalho, Mandibular and Hyoid Muscles of Galeomorph Sharks (Chondrichthyes : Elasmobranchii), with Remarks on Their Phylogenetic Intrarelationships. *Journal of Morphology* (2013) doi:10.1002/jmor.20166.
11. D. R. Huber, M. N. Dean, & A. P. Summers, Hard prey, soft jaws and the ontogeny of feeding mechanics in the spotted ratfish *Hydrolagus colliei*. *J. R. Soc. Interface* **5**, 941–953 (2008).
12. Anderson, P. S. L. Cranial muscle homology across modern gnathostomes. *Biological Journal of the Linnean Society* **94**, 195–216 (2008).
13. R. Diogo & J.M. Ziermann, Muscles of Chondrichthyan Paired Appendages: Comparison With Osteichthyans, Deconstruction of the Fore-Hindlimb Serial Homology Dogma, and

New Insights on the Evolution of the Vertebrate Neck: Muscles of Chondrichthyan Paired Appendages. *Anat. Rec.* **298**, 513–530 (2015).
14. J. M. Ziermann, , T. Miyashita & R. Diogo, Cephalic muscles of Cyclostomes (hagfishes and lampreys) and Chondrichthyes (sharks, rays and holocephalans): comparative anatomy and early evolution of the vertebrate head muscles. *Zoological Journal of the Linnean Society* **172**, 771–802 (2014).
15. A. L., Camp, B. R. Scott, E. L. Brainerd, & C. D. Wilga, Dual function of the pectoral girdle for feeding and locomotion in white-spotted bamboo sharks. *Proceedings of the Royal Society B: Biological Sciences* **284**, (2017).
16. M. I. Coates, K. Tietjen, A. M. Olsen, J. A. Finarelli, High-performance suction feeding in an early elasmobranch. *Science Advances* **5** (2019).
17. P.S.L. Anderson, M. Friedman, M.D. Brazeau & E.J. Rayfield. Initial radiation of jaws demonstrated stability despite faunal and environmental change. *Nature* **476** (2011)
